# A relatively rare traditional Chinese medicine pattern of primary Sjögren syndrome: A case report

**DOI:** 10.1097/MD.0000000000037744

**Published:** 2024-04-12

**Authors:** Qiang Chen, Xinru Chen, Yuqing Zhu, Xiali Yu

**Affiliations:** a Department of Neurology, The First Affiliated Hospital of Zhejiang Chinese Medical University (Zhejiang Provincial Hospital of Chinese Medicine), The First Clinical College of Zhejiang Chinese Medical University, Hangzhou, China; b The First Clinical College of Zhejiang Chinese Medical University, Hangzhou, China; c Department of Rheumatology, The First Affiliated Hospital of Zhejiang Chinese Medical University (Zhejiang Provincial Hospital of Chinese Medicine), Hangzhou, China.

**Keywords:** case report, primary Sjögren syndrome, traditional Chinese herbal medicine

## Abstract

**Rationale:**

This report presents a unique case of a patient diagnosed with Primary Sjögren’s syndrome and a relatively rare traditional Chinese medicine pattern, known as the combined cold and heat pattern and cold-dampness syndrome. The patient’s condition was successfully managed using Chinese herbal medicine, specifically the modified Da-Chai-Hu decoction and Linggui Zhugan decoction.

**Patient concerns:**

A 56-year-old woman had chronic dry eye and mouth for over 10 years. She was initially managed with traditional Chinese herbal medicine (TCHM) prescriptions, including the Zengye decoction, but the therapeutic effects were unsatisfactory. As the disease progressed, she was diagnosed with an anxiety disorder due to symptoms of vexation and insomnia. Treatment with alprazolam and venlafaxine failed to alleviate these symptoms. Recently, her general condition gradually worsened, with symptoms including a bitter taste in her mouth, dizziness, hot flashes, chills, poor appetite, chest discomfort, and constipation.

**Diagnoses:**

After a series of examinations, including a Schirmer test and labial gland biopsy, she was diagnosed with Sjögren’s syndrome.

**Interventions:**

Despite regular treatment with pilocarpine, sodium hyaluronate eye drops, venlafaxine, and alprazolam, the dry mouth symptoms intensified. Consequently, she sought further intervention through the TCHM.

**Outcomes:**

After 8 weeks of treatment with the modified Da-Chai-Hu decoction and Linggui Zhugan decoction, she reported a significant improvement in her dryness-related symptoms and sleep quality.

**Lessons:**

This case report demonstrates that TCHM can effectively treat Primary Sjögren’s syndrome, and should be considered for broader applications. Furthermore, this underscores the importance of tailoring treatment formulas to patients by identifying their specific syndrome differentiation in a clinical setting.

## 1. Introduction

Sjögren syndrome (SS) is a common chronic autoimmune disease that affects multiple organs and tissues.^[1]^ In most cases, clinical manifestations are characterized by persistent xerostomia and xerophthalmia, resulting from immune-mediated impairment of the salivary and lacrimal glands. Lymphocytic infiltration also occurs in the epithelial tissues of organs beyond the exocrine glands, leading to systemic symptoms, such as nephritis, bronchiolitis, muscle weakness, and pain.^[2,3]^ Notably, SS is reported to be present alone or in combination with other systemic autoimmune disorders, such as systemic lupus erythematosus, rheumatoid arthritis, and systemic sclerosis; thus, SS can be classified into primary SS (pSS) and secondary SS.^[4]^ Epidemiological data indicate that the prevalence rate of pSS is 0.03% to 5% worldwide, with a 1:9 male-to-female ratio, and that pSS occurs primarily at ages between 20 to 30 and 50 to 70 years.^[5,6]^ So far, although the etiology of pSS is largely unclear, genetic variants, environmental factors, infection, and family history may be implicated in the pathogenesis.^[5,7]^

Currently, the available treatment options are limited, mainly focusing on relieving symptoms, improving quality of life, and managing complications. Artificial tears and saliva secretion stimulators are the first-line treatments in clinical practice.^[8]^ Patients with systemic complications require immunosuppressants and/or steroid therapy to improve symptoms and reduce complications such as nephritis and joint symptoms.^[9]^ These drugs, however, have not been proven to be highly efficacious in the treatment of pSS.^[10]^ Additionally, the accompanying side effects of these drugs are difficult to ignore. Doctors would make a more targeted treatment decision in the clinical setting based on their own experience.^[10]^

Traditional Chinese herbal medicine (TCHM) is widely used to treat pSS in China.^[11,12]^ According to traditional Chinese medicine (TCM) principles, pSS belongs to the category of ‘‘dryness-bi” and most patients with pSS have hyperactivity of fire due to Yin deficiency, blood stasis, and phlegm stagnation.^[13]^ The treatments are to nourish yin, clear heat, remove blood stasis, and resolve phlegm. Various TCHMs have been reported for the treatment of pSS. This case report details the experience of a patient with pSS who exhibited a relatively rare combined cold and heat pattern, as well as cold-dampness syndrome. Despite persistent symptoms of dry mouth, dry eyes, chills, and constipation, and a poor response to first-line conventional drugs and Yin-nourishing treatment, the patient was successfully treated with a regimen involving the modified Da-Chai-Hu decoction (DCHD) and Linggui Zhugan decoction (LGZGD). However, this TCM pattern and treatment approach have not been previously reported. The aim of presenting and discussing this case is to share our insights with clinicians and offer an alternative treatment option for patients with similar conditions.

## 2. Case presentation

Herein, we describe the case of a 56-year-old female who visited our hospital in September 2021 with symptoms of dry eye and dry mouth for the past 10 years. The patient had visited several hospitals for treatment. However, no definite diagnosis could be made because of negative antinuclear antibody and rheumatoid factor test results. Oral TCHM prescriptions have been attempted, including the Zengye decoction (ZYD), which consists of *Radix scrophulariae, Ophiopogon japonicus*, and *Radix rehmannia*; however, the effects were not good. She denied a history of diabetes mellitus or insipidus. In addition, owing to the presence of vexation and insomnia, she was diagnosed with anxiety disorder by a physician from the mental health department and treated with venlafaxine and alprazolam for some time. She stopped taking this medication independently due to failed symptomatic relief. Over the past 4 months, the symptoms of dry mouth gradually worsened. Sleep was severely impaired, especially at night, accompanied by a bitter taste in the mouth, dizziness, hot flashes, chills, poor appetite, chest discomfort, and constipation. Therefore, the patient sought treatment again at the hospital.

**Figure 1.**
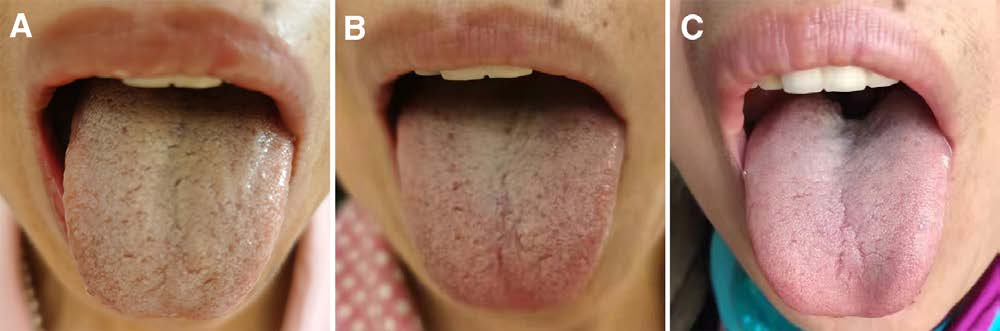
Images of tongue. (A) Before TCHM treatment. (B) After 5 d of TCHM treatment. (C) After 8 wk of TCHM treatment. TCHM = traditional Chinese herbal medicine.

**Figure 2.**
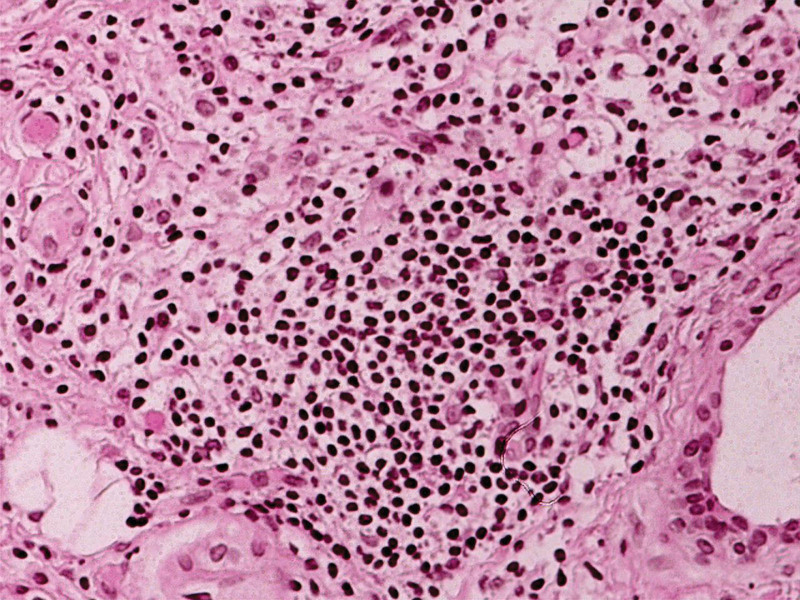
Hematoxylin-eosin (H&E) staining of labial gland (×200).

Physical examination showed that the patient had a stringy pulse, tender, and reddish tongue, and yellow greasy fur without rampant caries, joint swelling, and tenderness (Fig. 1A). Routine laboratory tests were performed and the results showed no apparent abnormalities, including blood routine, biochemistry, blood sugar, C-reactive protein, erythrocyte sedimentation rate, immunoglobulin G, immunoglobulin A, immunoglobulin M, rheumatoid factor, complement levels (C3 and C4), and tumor-associated markers. Notably, dry eye disease was evidenced by a positive Schirmer test result (left eye, 2 mm/5 min; right eye, 2 mm/5 min), which is a routine method for evaluating lacrimal gland function. Minor salivary gland biopsy revealed increased focal lymphocytic infiltration (>1 focus per 4 mm^2^ and approximately 200 lymphocytes per high power field) (Fig. 2). Magnetic resonance imaging of the head revealed normal findings. The throat mucosa showed obvious congestion on laryngoscopy, and laryngopharyngeal reflux and pharyngitis were suspected. According to the 2016 ACR/EULAR classification criteria for SS,^[14]^ the patient fulfilled the inclusion criteria with a weighted summed score of 4 points and was diagnosed with pSS.

**Figure 3.**
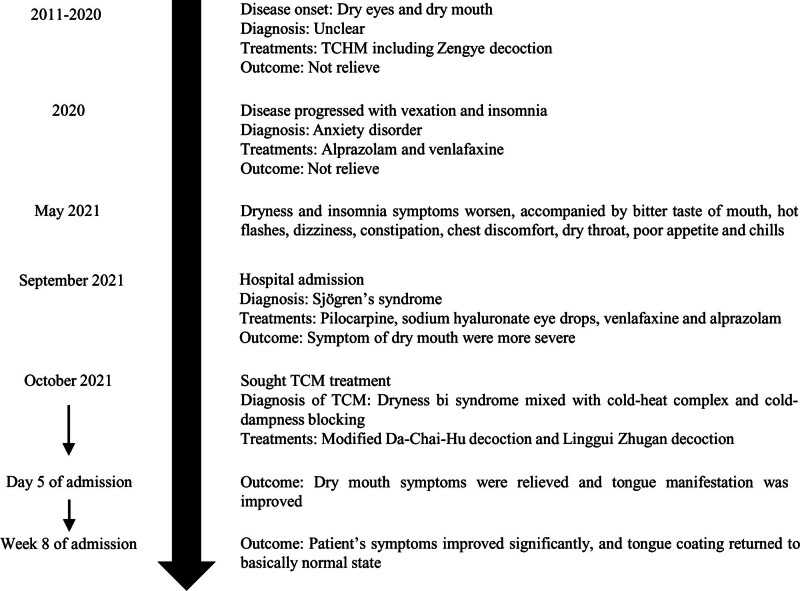
A timeline of disease progression and treatment. TCHM = traditional Chinese herbal medicine, TCM = traditional Chinese medicine.

First-line conventional treatments for pSS, including pilocarpine, sodium hyaluronate eye drops, and anxiolytics (venlafaxine and alprazolam), were prescribed to alleviate dryness and anxiety. However, this approach yielded no clinical benefits, and the patient’s dry mouth progressively worsened. Consequently, the patient sought help from the TCHM. According to the TCM theory, the patient exhibited a dryness bi syndrome mixed with a cold-heat complex and cold-dampness blockage. Based on the TCM syndrome type and physical findings, the patient was administered the modified DCHD and LGZGD (Table 1), and 1 pack of decoction (200 mL) was consumed twice daily. These treatments are known for their effects of warming Yang, invigorating the spleen, eliminating dampness, and relieving the exterior-interior syndrome. After 5 days of treatment, the patient’s dry mouth symptoms were noticeably relieved, and her tongue manifestations improved (Fig. 1B). After 8 weeks of treatment, the patient reported a significant improvement in her symptoms, with only slight dry mouth and chills remaining. The tongue coating returned to a nearly normal state (Fig. 1C). The timeline of disease progression and treatment is shown in Figure 3.

**Table 1:** The component of the modified Da-Chai-Hu decoction and Linggui Zhugan decoction.

Botanical name	Chinese name	Dosages (g/100 mL)
*Radix Bupleuri*	Chai-hu	2.25
*Rhubarb*	Da-huang	2.5
*Radix Paeoniae Alba*	Bai-shao	3.75
*Poria cocos*	Fu-ling	3.75
*Ramulus Cinnamomi*	Gui-zhi	2
*Atractylodes macrocephala*	chang Bai-shu	3.75
*Patchouli*	Guang-huo-xiang	2.25
*Artemisia annua*	Qing-hao	7.5
*Fortune Eupatorium*	Pei-lan	2.25
*Betelnut Peel*	Da-fu-pi	2.5
*Heterophylly Falsestarwort Root*	Tai-zi-shen	3.75
*Coix seed*	Chao-mi-ren	7.5
*Yam*	Shan-yao	3.75
*Finger Citron*	Fo-shou	2.25
*Coptis Rhizome*	Huang-lian	0.75
*Acorus gramineus*	Shi-chang-pu	2.5
*Reed Rhizome*	Lu-gen	11.25

## 3. Discussion

This report presents a case in which pSS was effectively managed with TCHM. The patient’s pSS-related symptoms were largely alleviated, and some clinical signs returned to normal. The use of the modified DCHD and LGZGD in treating pSS has not been previously reported. In this case, the efficacy of these decoctions in clinical practice was confirmed, providing further evidence of the unique advantages of the dialectical treatment system in TCM for managing complex diseases.

SS is a chronic progressive autoimmune disorder characterized by inflammatory infiltration of the exocrine glands, resulting in secretory dysfunction and serious complications including depression, fatigue, and systemic autoimmunity.^[3,15]^ Indeed, many inflammatory mediators and cells play a critical and complicated role in the pathogenesis of pSS, such as B cell-activating factor, a proliferation-inducing ligand, interleukin 12, interferon-γ, plasmacytoid dendritic cells, T cells, and B cells.^[10]^ For patients, once a clinical diagnosis is firmly established, the currently available treatment options for pSS, such as muscarinic agonists and immunomodulatory drugs, can only relieve symptoms and prevent acute exacerbation. However, it is not uncommon for these interventions to not achieve a satisfactory improvement in some cases, and these medications often inevitably cause serious side effects.^[3]^ Consequently, the search for more potent alternative drugs or combined strategies against pSS is necessary. Recently, many studies have shown that TCM can effectively improve the major symptoms of pSS and delay its progression by upregulating aquaporin proteins, inhibiting cell apoptosis, and preventing abnormal T and B cells activation.^[13]^ It suggests that TCM is a treasure trove for discovering novel drugs against pSS.

From the perspective of TCM, Yin deficiency syndrome is one of the most common syndromes in pSS patients. ZYD is a well-known TCM prescription for nourishing Yin in China, which can promote an increase in body fluids. However, in this case, the patient did not respond to ZYD because of a complex condition. The patient’s symptom-alternating fever and chills are manifestations of the struggle between antipathogenic qi and pathogenic factors outside and the inside. Moreover, vexation, bitter taste, and appetite are manifestations of heat in the gallbladder and stomach; heat and fluid deficiency correspond to dry throat; gallbladder heat corresponds to dizziness and dry eye; fullness in the chest and subcostal region points to Shaoyang disease; stasis due to exterior evil inward invade and transform to heat causes hot flashes and constipation, the symptom of Yangming disorder. The patient was diagnosed with Yangming and Shaoyang Syndrome Complex. Reddish tender tongue, thick greasy coating, and stringy pulse are signs of cold-dampness impediment. Thus, the patient had a cold-heat complex and cold-dampness impediment pattern. Meanwhile, the modified DCHD and LGZGD were used to release both exterior and interior, warm Yang, and transform dampness.

DCHD, a famous formula recorded in the “Treatise on Febrile Diseases,” is a classic formula for Yangming and Shaoyang Syndrome Complex through harmonizing and releasing Shaoyang and clearing interior excess heat. Previous studies have reported the efficacy of DCHD in the clinical treatment of various diseases, such as acute intrahepatic cholestasis,^[16]^ acute liver injury,^[17]^ cardiovascular disease,^[18]^ nonalcoholic fatty liver disease,^[19]^ pancreatic fibrosis,^[20]^ diabetes,^[21]^ gastrointestinal disease,^[22]^ mental illness^[23]^ by inhibiting inflammation, reducing oxidative stress, and improving lipid and glucose metabolism. Additionally, Zhongjing Zhang described LGZGD in the “Synopsis of Prescriptions of the Golden Chamber” (also named “Jingui Yaolüe”). It is widely used to warm the yang and fortify the spleen to move water. With the recent development of mass spectrometry and multiomics analysis techniques, modern pharmacological research has gradually uncovered the underlying molecular mechanisms by which TCM formulas with complex components in the treatment of various diseases. Multiple natural herbs from these formulas have anti-inflammatory, antioxidant, immune regulation, anticancer, antiaging, anxiolytic, and analgesic properties, including *Radix Bupleuri, Radix Paeoniae Alba, Rhubarb, Atractylodes macrocephala*, and *Poria cocos*,^[24–29]^ which may be the possible molecular mechanism in treating pSS.

It is worth noting that the patient had not yet suffered systemic organ damage despite suffering from the disease for nearly 10 years. Consequently, the patient had good prognosis in response to reasonable TCM prescription treatment. Further studies are required to determine whether similar therapeutic effects can be observed in the general population.

## 4. Conclusion

The modified DCHD and LGZGD show promise as standalone treatments for patients with pSS who exhibit a combined cold and heat pattern and cold-dampness syndrome. These herbal formulations could be viable treatment alternatives for patients with similar clinical conditions.

## Author contributions

**Conceptualization:** Xiali Yu.

**Investigation:** Xinru Chen, Yuqing Zhu.

**Project administration:** Qiang Chen.

**Writing—original draft:** Qiang Chen.

**Writing—review & editing:** Qiang Chen, Xiali Yu.

## References

[1] Lee AS Scofield RH Hammitt KM. Consensus Expert Panel (CEP) Members. Consensus guidelines for evaluation and management of pulmonary disease in Sjögren’s. Chest. 2021;159:683–98. 33075377 10.1016/j.chest.2020.10.011 PMC8438162

[2] Tarn J Lendrem D McMeekin P. Primary Sjögren’s syndrome: longitudinal real-world, observational data on health-related quality of life. J Intern Med. 2022;291:849–55. 35018685 10.1111/joim.13451 PMC9305875

[3] Negrini S Emmi G Greco M. Sjögren’s syndrome: a systemic autoimmune disease. Clin Exp Med. 2022;22:9–25. 34100160 10.1007/s10238-021-00728-6 PMC8863725

[4] Manfrè V Chatzis LG Cafaro G. Sjögren’s syndrome: one year in review 2022. Clin Exp Rheumatol. 2022;40:2211–24. 36541236 10.55563/clinexprheumatol/43z8gu

[5] Jin L Dai M Li C. Risk factors for primary Sjögren’s syndrome: a systematic review and meta-analysis. Clin Rheumatol. 2023;42:327–38. 36534351 10.1007/s10067-022-06474-8 PMC9873717

[6] Tarn J Lendrem D Barnes M. Comorbidities in the UK primary Sjögren’s syndrome registry. Front Immunol. 2022;13:864448. 35603172 10.3389/fimmu.2022.864448 PMC9116135

[7] Ma KS Wang LT Chong W. Exposure to environmental air pollutants as a risk factor for primary Sjögren’s syndrome. Front Immunol. 2022;13:1044462. 36865525 10.3389/fimmu.2022.1044462 PMC9972220

[8] Stefanski AL Tomiak C Pleyer U. The diagnosis and treatment of Sjögren’s syndrome. Dtsch Arztebl Int. 2017;114:354–61. 28610655 10.3238/arztebl.2017.0354 PMC5471601

[9] Ramos-Casals M Tzioufas AG Stone JH. Treatment of primary Sjögren syndrome: a systematic review. JAMA. 2010;304:452–60. 20664046 10.1001/jama.2010.1014

[10] Seror R Nocturne G Mariette X. Current and future therapies for primary Sjögren syndrome. Nat Rev Rheumatol. 2021;17:475–86. 34188206 10.1038/s41584-021-00634-x

[11] Liu H Wang X Liu W. Effectiveness and safety of traditional Chinese medicine in treatment primary Sjögren’s syndrome patients: a meta-analysis. Comb Chem High Throughput Screen. 2023;26:2554–71. 36959129 10.2174/1386207326666230322092252

[12] Wang W Wang X Fan Y. Complementary therapy with traditional Chinese medicine for a patient with Sjögren’s syndrome: a case report. Explore (NY). 2021;17:223–6. 32224257 10.1016/j.explore.2020.03.004

[13] Wei SJ He QM Zhang Q. Traditional Chinese medicine is a useful and promising alternative strategy for treatment of Sjogren’s syndrome: a review. J Integr Med. 2021;19:191–202. 33509710 10.1016/j.joim.2021.01.008

[14] Shiboski CH Shiboski SC Seror R. International Sjögren's Syndrome Criteria Working Group. 2016 American College of Rheumatology/European League Against Rheumatism classification criteria for primary Sjögren’s syndrome: a consensus and data-driven methodology involving three international patient cohorts. Ann Rheum Dis. 2017;76:9–16. 27789466 10.1136/annrheumdis-2016-210571

[15] Thalayasingam N Baldwin K Judd C. New developments in Sjogren’s syndrome. Rheumatology (Oxford). 2021;60:vi53–61. 34951923 10.1093/rheumatology/keab466 PMC8709567

[16] Xu S Qiao X Peng P. Da-Chai-Hu-Tang protects from acute intrahepatic cholestasis by inhibiting hepatic inflammation and bile accumulation activation of PPARα. Front Pharmacol. 2022;13:847483. 35370715 10.3389/fphar.2022.847483 PMC8965327

[17] Zhou Y Zhou Y Li Y. Targeted bile acid profiles reveal the liver injury amelioration of Da-Chai-Hu decoction against ANIT- and BDL-induced cholestasis. Front Pharmacol. 2022;13:959074. 36059946 10.3389/fphar.2022.959074 PMC9437253

[18] Yoshie F Iizuka A Komatsu Y. Effects of Dai-saiko-to (Da-Chai-Hu-Tang) on plasma lipids and atherosclerotic lesions in female heterozygous heritable Kurosawa and Kusanagi-hypercholesterolemic (KHC) rabbits. Pharmacol Res. 2004;50:223–30. 15225663 10.1016/j.phrs.2004.02.003

[19] Yang JM Sun Y Wang M. Regulatory effect of a Chinese herbal medicine formula on non-alcoholic fatty liver disease. World J Gastroenterol. 2019;25:5105–19. 31558860 10.3748/wjg.v25.i34.5105 PMC6747291

[20] Duan LF Xu XF Zhu LJ. Dachaihu decoction ameliorates pancreatic fibrosis by inhibiting macrophage infiltration in chronic pancreatitis. World J Gastroenterol. 2017;23:7242–52. 29142471 10.3748/wjg.v23.i40.7242 PMC5677205

[21] Zhang Z Leng Y Fu X. The efficacy and safety of dachaihu decoction in the treatment of type 2 diabetes mellitus: a systematic review and meta-analysis. Front Pharmacol. 2022;13:918681. 36003504 10.3389/fphar.2022.918681 PMC9393237

[22] Huang N Wei Y Liu M. Dachaihu decoction ameliorates septic intestinal injury via modulating the gut microbiota and glutathione metabolism as revealed by multi-omics. J Ethnopharmacol. 2023;312:116505. 37080366 10.1016/j.jep.2023.116505

[23] Horiba Y Yoshino T Watanabe K. Daisaikoto for menstrual pain: a lesson from a case with menstrual pain successfully treated with daisaikoto. Case Rep Med. 2015;2015:929514. 25792985 10.1155/2015/929514 PMC4352433

[24] Zhou Y Li QX Liao ZZ. Anti-inflammatory effect and component analysis of Chaihu Qingwen granules. J Ethnopharmacol. 2023;317:116763. 37315646 10.1016/j.jep.2023.116763

[25] Li QF Lu WT Zhang Q. Proprietary medicines containing *Bupleurum chinense* DC. (Chaihu) for depression: network meta-analysis and network pharmacology prediction. Front Pharmacol. 2022;13:773537. 35462897 10.3389/fphar.2022.773537 PMC9019785

[26] Yang L Wan Y Li W. Targeting intestinal flora and its metabolism to explore the laxative effects of rhubarb. Appl Microbiol Biotechnol. 2022;106:1615–31. 35129656 10.1007/s00253-022-11813-5

[27] Tung YT Chua MT Wang SY. Anti-inflammation activities of essential oil and its constituents from indigenous cinnamon (*Cinnamomum osmophloeum*) twigs. Bioresour Technol. 2008;99:3908–13. 17826984 10.1016/j.biortech.2007.07.050

[28] Zhu B Zhang QL Hua JW. The traditional uses, phytochemistry, and pharmacology of *Atractylodes macrocephala* Koidz.: a review. J Ethnopharmacol. 2018;226:143–67. 30130541 10.1016/j.jep.2018.08.023

[29] Bailly C. Atractylenolides, essential components of Atractylodes-based traditional herbal medicines: antioxidant, anti-inflammatory and anticancer properties. Eur J Pharmacol. 2021;891:173735. 33220271 10.1016/j.ejphar.2020.173735

